# The Texas Dental Law

**Published:** 1889-08

**Authors:** 


THE TEXAS DENTAL LAW.—A Bill to be entitled An Act to Regulate the Practice of Dentistry in the State of Texas.

*Be it enacted by the Legislature of the State of Texas:*

SECTION 1. That from and after the passage of this act it shall be unlawful for any person to engage in the practice of dentistry in the State of Texas, unless such person has obtained license from a board of examiners duly appointed and authorized by this act to issue such license; provided that dentists who have been in regular practice of dentistry in this State for three years next preceding the passage of this act shall not be required to submit to an examination and shall be entitled to a license without fee, which shall be transmitted to him by mail, otherwise upon his application accompanied by satisfactory evidence to the fact of his having been in the regular practice for the time required.

SEC. 2. That the board of examiners shall be appointed by the judge of each judicial district and shall be composed of three reputable dentists residing in said district, who shall hold their offices two years from the date of appointment, and any vacancy shall be filled by the district judge as aforesaid.

SEC. 3. The board shall, immediately after appointment, select one of their number as president and one as secretary, and adopt all rules necessary for the transaction of the business that may come before them.

SEC. 4. Said board shall meet annually at some central point in their respective districts to conduct examinations and grant licenses; notice of the time and place of such meeting shall be given for one month by publication in some newspaper published in the district.

SEC. 5. Any applicant who shall furnish satisfactory evidence of having graduated and received a diploma from a reputable dental college, and any applicants under the provision of the first section of this act; and all other applicants who undergo a satisfactory examination as to their qualifications, and shall pay to the said board a fee of five dollars, to be used for the advertising and incidental expenses, shall be granted license; which license shall entitle the person to whom granted to practice dentistry in any county when the same has been recorded as required by Section 12.

SEC. 6. Said board shall keep a book in which shall be registered the names of all persons licensed to practice dentistry by said board.

SEC. 7. The book so kept shall be a book of record, and a transcript from it, certified to by the officer who has it in keeping, with the common seal of said board, shall be evidence in any court in this State.

SEC. 8. That two members of said board shall constitute a quorum for the transaction of business, and should a quorum not be present on the day appointed for its meeting, the member present may adjourn from day to day until a quorum be present.

SEC. 9. That one member of said board may grant a license for an applicant to practice until the next regular meeting of the board, when he shall report the fact, at which time such temporary license shall expire, but such temporary license shall not be granted by a member of the board within one year after the board has rejected the applicant.

SEC. 10. That any person who shall in violation of the provisions of this act, practice dentistry in this State, for a fee or reward, shall be liable to indictment, and on conviction shall be fined not less than one hundred nor more than two hundred dollars; nor shall it be construed to prevent persons from extracting teeth, nor in any way interfere with physicians and surgeons in their practice as such.

SEC. 11. That all fines collected from prosecutions under this act shall be appropriated to the common school fund in the county where collected.

SEC. 12. That every person to whom license is issued by said board of examiners shall, within thirty days from the date thereof, present the same to the clerk of the county in which he resides, who shall officially record said license in a book in his office, and shall be entitled to demand a fee of fifty cents for his services, but a temporary license issued under Section 9, of this act, need not be recorded.

SEC. 13. That on the trial of any person indicted under the provisions of this act, it shall be incumbent upon the defendant in order to exempt him from the penalties of this act, to show that he has authority, under the law, to practice dentistry in this State.

SEC. 14. That all laws or parts of laws in conflict with this act, be and the same are, hereby repealed.

